# III-nitride tunable cup-cavities supporting quasi whispering gallery modes from ultraviolet to infrared

**DOI:** 10.1038/srep17970

**Published:** 2015-12-11

**Authors:** T. V. Shubina, G. Pozina, V. N. Jmerik, V. Yu. Davydov, C. Hemmingsson, A. V. Andrianov, D. R. Kazanov, S. V. Ivanov

**Affiliations:** 1 Ioffe Institute, St. Petersburg, 194021, Russia; 2 Linköping University, Department of Physics, Chemistry and Biology (IFM), Linköping, S-581 83, Sweden

## Abstract

Rapidly developing nanophotonics needs microresonators for different spectral ranges, formed by chip-compatible technologies. In addition, the tunable ones are much in demand. Here, we present site-controlled III-nitride monocrystal cup-cavities grown by molecular beam epitaxy. The cup-cavities can operate from ultraviolet to near-infrared, supporting quasi whispering gallery modes up to room temperature. Besides, their energies are identical in large ’ripened’ crystals. In these cavities, the refractive index variation near an absorption edge causes the remarkable effect of mode switching, which is accompanied by the spatial redistribution of electric field intensity with concentration of light into a subwavelength volume. Our results shed light on the mode behavior in semiconductor cavities and open the way for single-growth-run manufacturing the devices comprising an active region and a cavity with tunable mode frequencies.

Whispering gallery mode (WGM) microresonators have attracted growing attention as essential building blocks of nano emitters^1^,^2^,^3^, detectors, biological and chemical sensors^4^,^5^,^6^. They are especially advantageous for structures made from wide-gap III-nitrides^7^,^8^,^9^,^10^,^11^, because manufacturing the planar Bragg microcavities is difficult with decreasing the thicknesses of constituent layers in the ultraviolet (UV) range. Such a problem is not essential for a WGM microresonator, since the working wavelength determines the characteristic size of its body as a whole. On the other end of the spectral range, the narrow-gap III-nitrides could cover the near infrared (NIR) range corresponding to the minimal losses in telecommunication networks. However, the structural quality of these materials is usually not too good. Thus, the challenge still remains of fabricating the WGM cavities from all the III-nitrides which have good prospects for nanophotonics in general.

The emergence of narrow lines in the optical spectra of the WGM microresonators is usually associated with the Purcell effect^12^,^13^,^14^, when the spontaneous emission probability of a dipole resonantly coupled to a cavity mode is enhanced by a factor 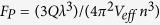. Here *λ* is the wavelength in free space, *n* is the refractive index of the cavity material, and 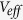 is the effective volume of the mode, which gives the level of its confinement. The quality (*Q*) factor of a particular resonance is defined as 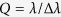, where 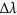 is the width of the WGM-related narrow line. It is commonly believed that the Purcell effect always plays a positive role of increasing the emission efficiency, that is not unconditionally valid. As demonstrated by Kleppner^15^, the inhibition of spontaneous decay rate by a factor of order 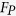 can take place when the density of final photon states or cavity size are too small. Similar effect appears with detuning from the mode resonance frequency^16^.

The ratio *γ* of spontaneous emission rate of an emitter having the wavelength *λ*_*e*_ located at position *r* in the cavity to the rate in a homogeneous medium can be expressed as 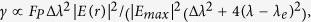 neglecting the decay channel into leaky modes^16^,^17^. Here the electric field amplitude distribution in the cavity 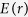 has a maximum magnitude 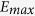. This equation shows that to achieve noticeable enhancement the mode resonance should be tuned to the emitter resonance and the emitter itself should be placed to a region where the mode with high *Q*-factor is strongly confined. We assume that the distribution of electromagnetic mode intensity in a WGM cavity, which is spatially inhomogeneous in principle, will control the local manifestation of the Purcell effect via either enhancement or inhibition of emission. Little attention was paid to this fact in the investigations of intensity distribution in the WGM cavities^18^,^19^.

Currently, there is a need for a cavity design which satisfies three basic requirements: i) high *Q*-factor, ii) tunability of working frequencies, and iii) compatibility with the chip technology. Operation of a dielectric cavity with high *Q*-factor, when the WGMs are strongly confined inside, requires either special scatters or elements for evanescent coupling^20^,^21^. This problem is diminished in the semiconductor cavities by using the fluorescence coupling^5^. For that, an emitting substance (impurities, quantum wells or dots) is inserted within the resonator^7^,^9^. Under the illumination by a short-wavelength light, the broadband emission of this substance appears at longer wavelengths, below the principal absorption edge of cavity material. This emission launches the WGMs and couples to them. The light out-coupling, being weakly dependent on cavity orientation^18^, occurs mostly via the boundary-wave and pseudointegrable leakages^22^. It is worth mentioning that the *Q*-factor can hardly be very high in the “open” cavities emitting light. However, it is not a fatal shortcoming, since the resonators of the moderate quality might be quite useful for many nanophotonics applications. To tune WGMs in the cavities, there are a few methods: temperature^23^, strain^24^, and shape variation, like in the so-called “bottle microresonators”^25^. In particular, the temperature-induced shift at the optical pumping reached 3.7 meV in a hybrid structure comprising a nanoparticle and a glass microsphere^26^.

In many cases, the WGM cavities are formed by post-growth etching^7^,^8^,^9^; alternatively, they can be created by epitaxy^17^,^27^,^28^. Both approaches possess inherent advantages and disadvantages. The etching allows fabricating structures of large diameters, which can maintain the long-lived high order modes with enhanced *Q*-factor. However, the number of modes existing in the vicinity of the principal one increases^29^, that hampers the spectral selection of discrete transitions. The rotational symmetry of the etched discs complicates the light into- and out-coupling. Epitaxial techniques offer a nice opportunity to fabricate a cavity and an active region during a single growth run. The formed cavities are nano- and microcrystals, which can be precisely located on the growth surface^30^. Their sizes are more suitable for supporting the low-order modes^31^ that facilitates the spectral selection. On the other hand, these cavities have usually a shape which reproduces the crystal structure, i.e. hexagonal in III-nitrides and II-oxides. Distortion of this shape may unpredictably change the WGM frequencies^27^.

Here, we present a novel type of microresonators —a cup-cavity, which can be used in a wide spectral range from UV to NIR. The monocrystal cup-cavities were fabricated by molecular beam epitaxy (MBE) from GaN and InN to prove applicability of our approach for the whole III-nitride family. The cup-cavities support the so-called quasi-WGMs of low orders, whose frequencies and electric field intensity distribution deviate from those in discs. The impact of the WGMs on the cavity properties is justified by comprehensive investigations done in a wide temperature range by using high-spatial-resolution spectroscopy and imaging techniques and supported by theoretical modeling. The tunability of the quasi-WGMs is conditioned by strong optical dispersion in semiconductors materials near an absorption edge. We report on the discovered effect of mode alteration (switching), which is accompanied by the modification of spatial mode intensity distribution and emission of terahertz (THz) quanta. In some degree, the cup-cavity operates as a parabolic mirror concentrating the light. At room temperature, the mode energy is confined into a subwavelength volume, that can be used for selective enhancement of a limited number of quantum emitters.

## Results

### Samples

**Figure 1:**
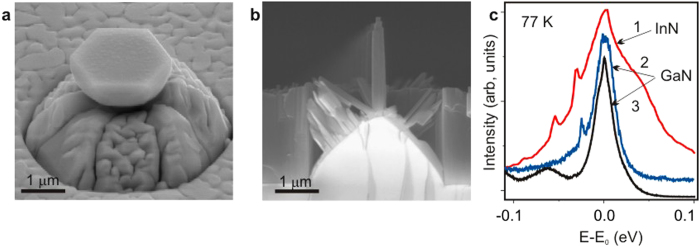
Typical SEM images and *μ*-PL spectra of monocrystals grown by MBE on cone-shaped patterned substrates. (**a**) An InN cup-cavity, (**b**) a GaN nanocolumn array shown from the cleaved facet of a sample. (**c**) *μ*-PL spectra measured in: 1) the InN cup-cavity, 2) the GaN cup-cavity, and 3) the GaN nanocolumns. The spectra are normalized and arbitrarily shifted for the sake of demonstrativeness; the energy counts off from the PL peak energy E_0_ which is taken as 0.758 eV for InN and 3.472 eV for GaN. The narrow lines are absent in the nanocolumn spectrum because their small diameter does not support the WGM modes.

Our cup-cavities were grown on cone-shaped patterned *c*-sapphire substrates by MBE which has been developed to create the site-controlled monocrystals of different shapes, such as cups and nanocolumns (Fig. 1). Note that the latter are promising for single-photon emitters^30^. Details on the MBE technology for both GaN and InN monocristals are given in the Methods and illustrated by scanning electron microscopy (SEM) images in Supplementary, Fig. 1. The metal-rich conditions turn out to be preferable for the cup-cavity formation. The creation of the InN cup-cavity was a challenge, since InN is the most problematic material for application among III-nitrides. Importantly, the wide band of near-band-edge emission in InN is very suitable to depict the almost full set of anticipated modes by means of the fluorescence coupling. This would be impossible using the GaN crystals, where the PL line is typically narrow and can coincide only with 1–3 modes (Fig. 1(c)). As a result, most of the results presented below concern the InN cup-cavities.

**Figure 2:**
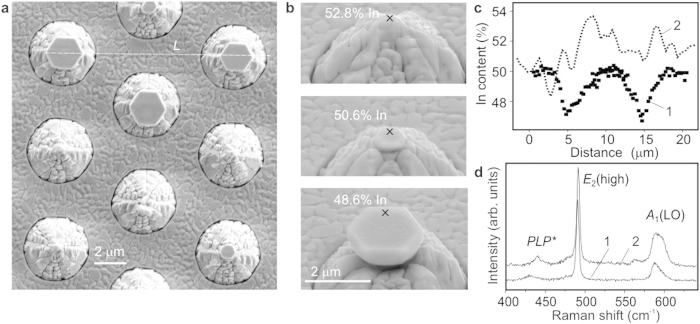
Improved structural properties of InN monocrystals as compared with planar layers. (**a**) A plane-view SEM image showing smoother top surface of the crystals than the surface of surrounding planar areas. (**b**) Evolution of the crystal size and composition during growth. The points of EDX microanalysis are marked by ‘x’; the In content is given nearby. (**c**) The EDX profiles of In content done: 1—along a line *L* in (**a**); 2—across the area enriched with metallic indium in a reference planar layer. The large crystal cavities are characterized by some lack of In. (**d**) First-order *μ*-R spectra taken from: 1—the crystal, 2—the planar layer. The crystals exhibit the narrower widths of allowed E_2_ and A_1_(LO) Raman modes and the shift of a mixed plasmon-LO-phonon mode PLP* towards lower frequencies.

The structural properties of the cup-cavities were investigated by SEM, energy dispersive x-ray (EDX) microanalysis, and micro-Raman (*μ*-R), whose details are given in Methods, as well as the details of spectroscopy and imaging techniques. According to SEM studies, the crystal surface is smoother than that of a surrounding area (Fig. 2(a)). A significant part (~20%) of the monocrystals possesses the ideal shape and large characteristic diameter (*d*). Such a yield is sufficient for practical applications; moreover, it can be further improved. Currently, the maximal *d* *~* 2.2 *μ*m achieved for the InN cup-cavities exceeds that for the GaN ones (~1.2 *μ*m). However, in both cases the low-order optical modes can be supported, since the light wavelength inside GaN (~0.12 *μ*m) is much smaller than that in InN (~0.55 *μ*m).

The crystals fabricated on the patterned substrate exhibit better structural quality than a reference layer grown using the identical MBE regimes on a planar substrate. In accordance with previous investigations^32^, the Raman data shown in Fig. 2(d) correspond to the lower densities of structural defects and decreased concentration of free carriers. In general, the InN tends to non-stoichiometry and spontaneous formation of metallic In nanoparticles^33^,^34^. Plasmonic resonances in such nanoparticles could enhance emission in a semiconductor matrix nearby or induce additional losses, depending on their sizes^35^. When considering the amplification of the emission intensity in our cup-cavities, we should exclude the plasmonic enhancement as a possible mechanism, because no signs of In excess were found in the large InN crystals (Fig. 2(b,c)).

**Figure 3:**
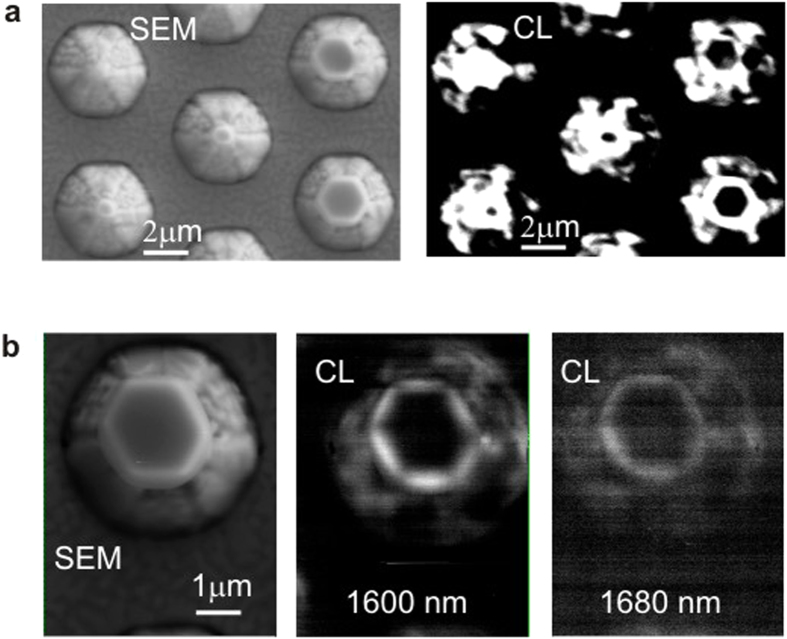
SEM and *μ*-CL images (orthogonal view) recorded at 5 K from an InN sample with the cup-cavities using different CL regimes. (**a**) SEM and panchromatic CL images of the same area with the crystals of different sizes. (**b**) SEM and mono-CL images recorded from the same single crystal with the central wavelengths of detection: 1600 nm (0.775 eV), and 1680 nm (0.738 eV).

**Figure 4:**
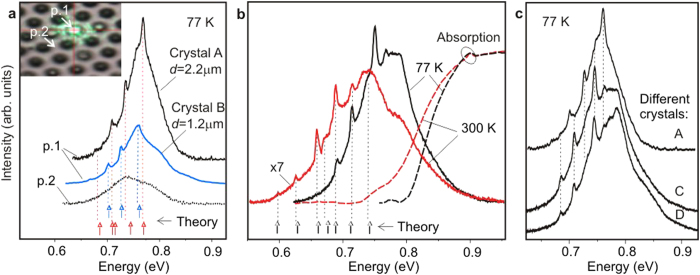
WGM-related narrow lines in *μ*-PL spectra measured in InN structures. (**a**) The inset demonstrates the spot of a laser beam (532 nm), which can be focused either on the top surface of a crystal (p. 1) or on the area between the cones (p. 2). No narrow lines were observed in the latter case. The energies of the narrow lines are not identical in the crystals of different sizes: A (*d* = 2.2 *μ*m) and B (*d* = 1.2 *μ*m). (**b**) *μ*-PL spectra of the crystal C (*d* = 2.2 *μ*m) measured at 77 K and 300 K, shown together with integral absorption spectra (dashed lines). The narrow line energies at a distance from the absorption edge are identical at different temperatures. Theoretical energies are marked at the bottom axes in (**a**,**b**) (see text for details). (**c**) *μ*-PL spectra measured in different crystals with 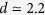 *μ*m, exhibiting the similar energies of the narrow lines.

Micro-cathodoluminescence (*μ*-CL) studies showed that the brightest emission spots are always at the crystals (Fig. 3(a)) that is consistent with their good material quality. However, this emission is not homogeneous, but reflects the spatial distribution of electromagnetic energy inside the cavities. At low temperatures, the smaller the crystal size, the higher the intensity of emitted light. Such a behavior is consistent with the pseudointegrable light out-coupling^22^, which is proportional to 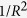. In the mono-CL images (Fig. 3(b)), the brightest emission patterns were recorded at the energies of the strongest narrow lines observed in the micro-photoluminescence (*μ*-PL) spectra (Fig. 4). At the high-energy side of these spectra, where the narrow lines are absent, the emission from the crystals merges into the background emission from the surroundings.

When the temperature increases, the emission from the small crystals quenches fast due to their low cavity quality, while in the large ones it can be detected up to room temperature. The average decay time of emission is about 150 ps at low temperature (10 K). This time was considerably shortened (up to 90–100 ps) to 100 K when the radiation of small crystals was fully quenched. Further increase in temperature did not affect markedly the decay time. One can consider this shortened decay time as characteristic of the large crystals which still emit light. In this case, it can be ascribed in part to the acceleration of the recombination rate due to the Purcell effect.

### Quasi whispering gallery modes

The *μ*-PL spectra measured in the InN cup-cavities comprise narrow WGM-related lines superimposed on a wide emission band (Fig. 4). Such lines are absent with measurements from the planar area (Fig. 4(a)). The distinctiveness and strength of these narrow emission lines are comparable with those in the disc resonators fabricated by etching^7^,^9^. Around PL band maximum, the free spectral range (FSR) varies from 13 meV to 30 meV, that allows the selective enhancement of optical transitions. Note that the conventional expression for FSR evaluation in discs^7^ is not applicable to the cup-cavities due to the more complicated pathways of light in them. The narrow-line widths are weakly varied across the InN emission band in different crystals. Taking the widths from the *μ*-PL spectra, the *Q*-factor is estimated to be in the ranges 170–200 and 700–900 for the modes in the InN and GaN cup-cavities, respectively. The reduced values for the InN cavities are in part due to the lower spectral resolution of the NIR measurements.

In the cup-cavities, the narrow emission lines are observed at the maximum and lower-energy part of the emission spectra. At the higher-energy side they are suppressed, mainly because of optical losses induced by the close absorption edge. With a temperature rise, the broadened absorption edge quenches those of the modes, which were at the maximum. Instead, the new modes come into play at the lower-energy side of the shifted PL band, because of changing the fluorescence coupling conditions. Between these boundaries, a set of the narrow lines can be found, whose positions are stable with increasing the temperature from 77 K to 300 K (Fig. 4(b)). It is essential that the narrow lines have insignificant broadening in such a wide temperature range.

The amazing finding is the observed identity of the mode frequencies recorded in all ‘ripened’ crystals of the largest size, which were chosen from the different parts of a wafer (Fig. 4(c)). This identity, existing within the limits of experimental accuracy, means that variation in shape and characteristic sizes of the large microcrystals is negligible. Such a similarity implies that the self-limitation (saturation) of the crystal sizes takes place at the prolonged MBE growth. Note that the standardized mode frequencies are a prerequisite for any cavity application.

### Mode switching

**Figure 5:**
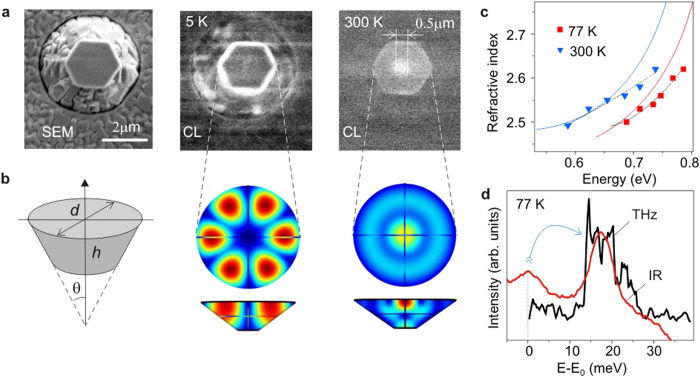
Temperature-induced mode switching in the cup-cavities. (**a**) SEM and *μ*-CL images (orthogonal view) of the same InN crystal recorded at 5 K and 300 K showing the change of the dominant mode type from azimuthal to radial. (**b**) The simulated distributions of electromagnetic field intensity, as seen in the plane and cross-section of the crystal, shown together with the schematic shape used in these simulations. (**c**) Variation of the refractive index derived from the fitting of the WGMs at low (squares) and room (triangles) temperatures; the dotted lines is their polynomial fitting; the solid lines indicate the index dependencies derived from the integral optical spectra. (**d**) Teraherz spectrum shown together with the peak of the NIR emission comprising two unstable WGM-related lines. The energy scale is taken as 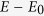, where 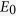 is the peak energy of the first line for the NIR emission, while for the THz band 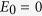.

The remarkable effect of mode switching accompanied by changing the spatial mode intensity distribution was discovered by the *μ*-CL measurements performed in the 5-300 K range. At low temperatures, the spatial distribution of the *μ*-CL signal corresponds to the azimuthal type, when the highest density of electromagnetic field is at the periphery of a crystal. Such an emission pattern can be observed even in the rather small crystals (Fig. 3(a)). When the temperature increases up to 100-150 K, the emission pattern starts to change. At room temperature, it corresponds to the radial type when the most bright spot occurs in the center of the crystal (Fig. 5(a)). To shed light on this phenomenon, let us remind that in the simplest cylindrical approximation the frequency of a resonator mode of the *m*-order obeys the law 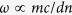, where *c* is the speed of light and *m* is integer. Therefore, with the constant geometry, i.e. when the same crystal is measured and the small thermal expansion of its size can be neglected, only variation of the refractive index can change the type of the modes with increasing the temperature. The variation of the absorption coefficient will rather suppress the modes, as it was observed at the higher-energy side of the *μ*-PL spectra.

We have performed the numerical simulation of the quasi-WGMs in the cup-cavities to prove the anticipated effect of the refractive index variation. The modeling has been done by solving the Maxwell’s equations with appropriate boundary conditions, which is the most general way to search the modes in optical microcavities^36^. The modeling procedure included the fitting of experimental mode energies taken from the *μ*-PL spectra. The comparison of the theoretical and experimental energies is presented in Fig. 4(a) for crystals of different sizes and in Fig. 4(b) for different temperatures. The examples of the spatial distributions of the electromagnetic field intensity inside the cavities at different temperatures are depicted in Fig. 5(b). The extended sets of such distributions for both InN and GaN cavities are shown in the Supplementary, Fig. 2.

To determine the refractive index dispersion dependencies, needed for these simulations, we measured first the integral absorption and reflection at different temperatures; then, the complex dielectric function was extracted by applying the Kramers-Kronig relations. The obtained dependencies, being reasonably consistent with published data^37^,^38^, were used in our preliminary calculations. More accurate analysis was done using the refractive index as a fitting parameter. Namely, its value was varied to search a feasible mode in the vicinity of an experimentally observed narrow line. Figure 5(c) shows the refractive index values derived from such simulations. They deviate noticeably from the dependencies (solid lines) obtained by the analysis of the integral optical spectra taken from areas comprising both the crystals and planar layer. This difference arose because an effective absorption edge in an InN layer can shift towards lower energies due to the optical losses induced by the metallic In nanoparticles, while the shift in the opposite direction may occur when the material is In-depleted^34^. Bearing that in mind, we can conclude that the modeling of the modes represents a unique way to determine the optical parameters characteristic for the crystal material itself.

For the GaN cavities, the modeling has confirmed that the number of the modes within the emission band is limited: only three distinct modes were found. The anticipated mode at 3.450 eV coincides reasonably with the narrow line at 3.447 eV in the experimental spectrum shown in Fig. 1(c). We assume that the two other modes, found near 3.47 eV, can be suppressed by neutral-donor bound exciton scattering which provides the strong attenuation of propagating light in GaN^39^. However, the basic result of the modeling is the evidence that the azimuthal type of quasi-WGMs prevails over the radial type at low temperatures in the InN cup-cavities and that this situation changes to the radial type at room temperature. Notably, the variation of the refractive index in semiconductor structures may be achieved under pumping by the laser beam or electric pulses. Moreover, these methods can change the concentration of free carriers, which in turn affects the refractive index to a considerable extent. At the optical pumping, likely, the combination of these factors induced the mode intensity variation at the PL maximum in the spectra shown in Fig. 4(c), which were measured at somewhat different laser beam focusing conditions.

We assume that the switching between two neighboring modes has to gain the small energy excess, which can radiate as the quanta of terahertz (THz) emission. Such an assumption is well consistent with the results of THz measurement performed using electric-pulse excitation technique^40^,^41^. As demonstrated, the electric pumping of InN structures induces the similar broad NIR emission band^42^, which can launch the quasi-WGMs via the fluorescence coupling. In the spectrum of THz emission shown in Fig. 5(d), the peak energy at 14 meV (~3.5 THz) corresponds well to the energy separation (13–15 meV) between the adjacent modes at the maximum of the IR emission band. Note that the modes are absent at the higher-energy side of the band and that their separation increases up to 20–30 meV at the low-energy wing. Such a situation may be a reason for a strongly asymmetrical shape of the THz spectrum.

The used excitation regime by the well-separated packets of electric pulses (see Methods) was optimized to prevent the overheating of the samples. Therefore, we assume that the increase in the carrier concentration governs the mode switching process. If the carrier concentration is increased at the electric-pulse propagation in the realistic range of 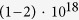 cm^−3^, the modification of the real part of the complex refractive index will comprise several percents from an initial value^42^, which can dramatically modify the conditions of the maintenance of modes. Other mechanisms, including nonlinear ones, should provide the THz emission in InN at different energies^41^. In particular, such emission could arise due to the surface plasmons coupled to electromagnetic field at the Bragg grating formed by the cones on the patterned substrate. However, for the used cone period the emission frequency should be below 2 THz, i.e. significantly less than the measured frequency of the THz signal in the InN structure with the cup-cavities.

## Discussion

We have demonstrated the novel cup-cavities operating in principle from UV to NIR, which were fabricated by MBE from III-nitride semiconductors — GaN and InN, without any post-growth treatment. Realization of these cup-cavities evidences the potential of MBE for the cavity nanotechnology. Note that up to now there was a lack of nanophotonic devices based on InN, although this semiconductor has been intensively studied since the beginning of this century. Therefore, successful fabrication of the InN cup-cavities with stable mode frequencies and pronounced emission enhancement is a certain technological breakthrough in this field. The cavities exploit the mechanism of fluorescence coupling by their own near-band-edge emission, which allowed us to discover new effects emerging near an absorption edge in semiconductor cavities such as temperature-induced mode alteration and energy confinement on a subwavelength level.

We highlight that the change of the refractive index, controlling the quasi-WGMs, can be realized not only by the temperature increase, but also by laser beam and electric-pulse pumping. In contrast with the slow temperature exposure, their heating effect is fast. Furthermore, these techniques change the free carrier concentration. In a degenerate semiconductor, like InN, it provides the Burstein-Moss effect which will push the apparent absorption edge towards higher energies due to the filling of conduction band states. Therefore, its impact on the complex refractive index will be opposite to the temperature-induced red shift of the absorption edge. In this case, two scenarios of the mode alteration can be realized. First, the energy of a mode will be increased by few meV with the conservation of its spatial intensity distribution, that might be useful for fine adjustment to a resonance in a nano-emitter. Alternatively, the abrupt switching to the neighboring mode, promising for optical switchers, can take place with changing the intensity distribution and emitting the THz quanta. In our studies, the weighty argument in favor of the later is the registration of the THz emission.

Considering the emission intensity pattern in the low-temperature *μ*-CL images of the crystals (Figs 3 and 5(a)) we should draw attention to the strong contrast between the emitting periphery and dark central area, which reflects the difference in emission dynamics within these regions. In the local areas where the density of photon states determined by the electric field intensity, 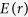, is close to zero, the emission is strongly inhibited, although the mode Purcell factor 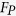 can be high. At room temperature, the mode energy is confined in a small subwavelength volume in the center of the cavity (Fig. 5(a)). Its characteristic size ~500 nm is about 1/3 of the free-space wavelength *λ*, that is well consistent with the anticipated smallest possible electromagnetic mode volume 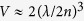, achievable in a dielectric cavity^44^. Such mode confinement can be used to select the limited number of radiating dipoles in a semiconductor cup-cavity instead of mesa formation by expensive electron-beam lithography.

In summary, we propose the III-nitride cup-cavities exploiting quasi-WGMs with *Q*-factor approaching 900 in the UV range, that is sufficient for enhancement of the nano-emitter efficiency and detection sensitivity. Small electromagnetic mode volume allows spatial selection of radiating dipoles in addition to spectral selection by the narrow mode lines. Furthermore, we show that the WGMs in a semiconductor cavity can be controlled by the complex refractive index variation near the absorption edge. This opens a way to realize the semiconductor WGM cavities with tunable frequencies controlled by optical or electric-pulse pumping.

## Methods

### Molecular beam epitaxy growth

III-nitride monocrystal cavities were grown by the low-temperature plasma—assisted (PA) MBE on cone-shaped patterned sapphire substrates using a Compact 21T (Riber) setup equipped with a nitrogen plasma activator HD25 (Oxford AR). The substrates have the regularly positioned cones with a diameter and height of 3 and 1.5 *μ*m, respectively. These cones act as pedestals for the grown monocrystals. The formation of the crystals on their tops is mainly induced by the difference in growth rates along basic crystallographic directions, stimulated by chosen technological parameters. The important factors which control the crystal shape are the substrate temperature 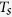, fluxes ratio 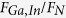, and polarity, N or Ga(In), which is determined by a buffer layer. Their variation can change the shape of the monocrystals from inverted hexagonal pyramids to nanocolumns. The procedure of their fabrication comprises consecutive growth of the thin buffer layer and basic micrometer-sized layer at different parameters (Table 1). For the buffer growth, either the ordinary MBE or migration enhanced epitaxy (MEE) was exploited. The growth procedures are illustrated by SEM images given in Supplementary Fig. 1.

### Measurements

The *μ*-PL studies were carried out at 77 and 300 K using Horiba Jobin Yvon T64000 and LabRAM HR spectrometers equipped by a Linkam THMS600 temperature-controlled microscope stage. The measurements were done with cw excitation by 532 nm and 325 nm laser lines for InN and GaN structures, respectively. We exploited the objective Mitutoyo 50x UV (NA = 0.40) and a liquid nitrogen-cooled charge-coupled detector (CCD) to measure the GaN cavity spectra with the spectral resolution of about 0.5 meV. The Mitutoyo 100x NIR (NA = 0.50) objective and an InGaAs photodiode with a thermoelectric cooler was used to measure the InN cavity spectra with the spectral resolution of about 1.0 meV. The beam impinging normally to the surface was focused by the objective into a spot with FWHM of 1 *μ*m that is enough to measure separately the monocrystals. The same objective collected the PL signal from sample surface. The *μ*-R measurements were done using the same setup at 300 K. The spectra of transmission and reflection were measured using a two-monochromators setup with excitation by a tungsten lamp. The time-resolved PL measurements were performed in a closed-cycle He cryostat using a 710 nm line of a pulsed laser for excitation. These integral cw and time-resolved spectra characterize the ~1-mm-sized areas which include both the crystals and surrounding layer.

SEM, microanalysis, and *μ*-CL studies were done using a LEO 1550 Gemini analytic scanning electron microscope equipped by a unit for EDX and a low-temperature *μ*-CL stage with a liquid nitrogen-cooled Ge-detector. At the EDX characterization performed with 0.5% accuracy, the nitrogen calibration was done using a perfect bulk GaN sample. The *μ*-CL studies were carried out at 10 kV beam voltage from 5 K up to 300 K with signal collection normally to the top monocrystal surface. Both panchromatic and mono-CL regimes were used. For the later, a CL signal was registered at particular mode wavelengths within the emission band.

The THz spectra were measured at 77 K with excitation by the series of packets of rectangular pulses with 15 V pulse height, 10 *μ*s duration, and 71.5 Hz reputation rate, as described in^40^,^41^. Wide-angle detection was done normal to the sample surface. The pulses passed through contacts formed on the top surface of a ~5-mm-long sample. Such a pulsed excitation prevents the heating of a sample. The step-scan Fourier spectrometer has the volume of optical path evacuated down to 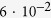 Torr to exclude any influence of water vapor absorption on the shape of the THz spectra.

### Modeling

The spatial distribution of the electromagnetic field intensity inside the cavities and mode frequencies were searched for the quasi-WGMs existing within the limits of emission bands of the cavity materials. It was done numerically by solving the Maxwell’s equations using the Comsol Multiphysics software. We adopted boundary conditions so as to cause a rapid decay of light waves outside the crystal. The crystal dimensions were taken from the SEM data. In these simulations, the cup-like shape of the cavities was approximated by a truncated cone. For GaN crystals, the prism-like shape was taken as more appropriate. In this case, we revealed the distinct slab (Fabry-Pérot) modes propagating between two parallel facets in the crystals.

## Additional Information

**How to cite this article**: Shubina, T. V. *et al.* III-nitride tunable cup-cavities supporting quasi whispering gallery modes from ultraviolet to infrared. *Sci. Rep.* **5**, 17970; doi: 10.1038/srep17970 (2015).

## Supplementary Material

Supplementary Information

## Figures and Tables

**Table 1:** Typical parameters of MBE growth used for InN and GaN monocrystal fabrication.

Structure	Buffer	Buffer growth parameters	Basic growth parameters
InN cup-cavities	200-nm-thick GaN	MBE, 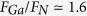 ,*T*_*s*_ = 700 °C	MBE, 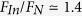 ,*T*_*s*_ = 470 °C
GaN cup-cavities	70-nm-thick AlN	MEE, 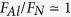 ,*T*_*s*_ = 784 °C	MBE, 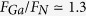 *T*_*s*_ = 650 °C
GaN nanocolumns	40-nm-thick GaN	MBE, 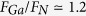 ,*T*_*s*_ = 605 °C	MBE, 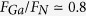 ,*T*_*s*_ = 750 °C
