# Supplementary material for: III-nitride tunable cup-cavities supporting quasi whispering gallery modes from ultraviolet to infrared
Source: Sci Rep. 2015 Dec 11;5:17970. doi: 10.1038/srep17970 (PMC4675966; doi:10.1038/srep17970)
Supplement: Supplementary Information [file srep17970-s1.pdf]

SUPPLEMENTARY INFORMATION

for

III-nitride tunable cup-cavities supporting quasi whispering gallery modes from ultraviolet to near infrared

by

T. V. Shubina, G. Pozina, V. N. Jmerik, V. Yu. Davydov, C. Hemmingsson,

A. V. Andrianov, D. R. Kazanov, and S. V. Ivanov

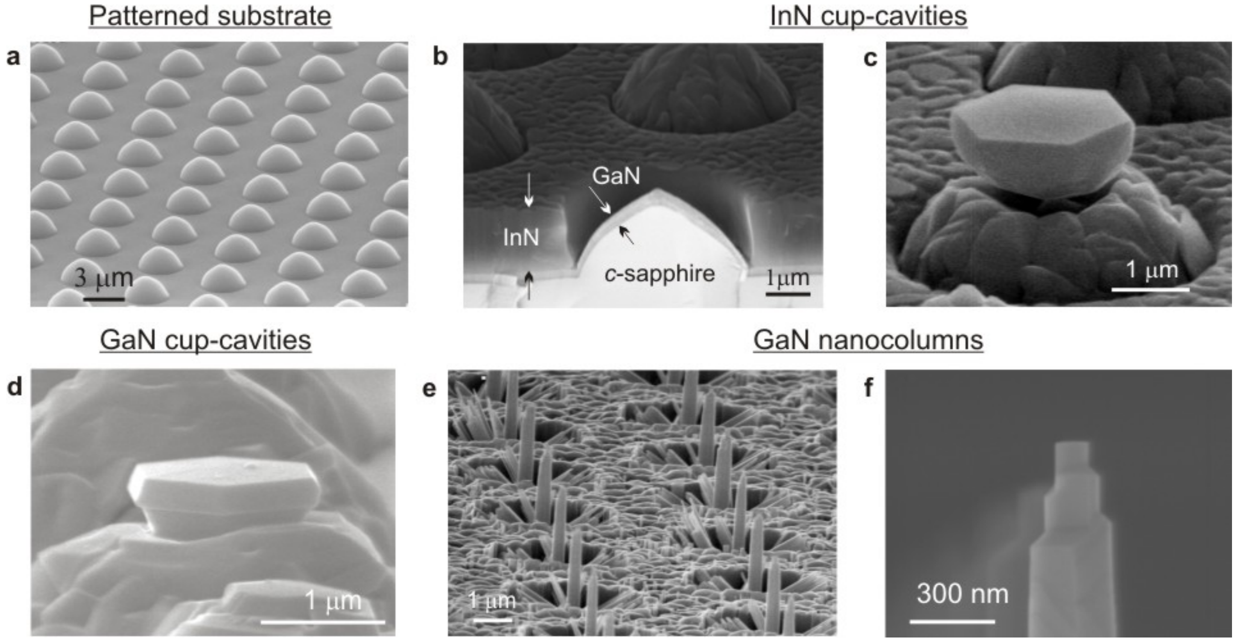

FIG. 1. SEM images illustrating the MBE growth of III-nitride monocrystals. (a) Cone-shaped patterned sapphire substrate. (b) Cleaved edge of a sample with an InN layer grown on the substrate above a GaN buffer. The basic InN layer was grown during ~3 hours, that results in the nominal layer thickness of ~1.5 μm. (c) Overhead view on an InN cup-cavity formed on the top of a cone. (d) The GaN cup-cavity. (e) Array of regularly situated GaN nanocolumns. The nanocolumns arise from a crater, because the growth on the cone tops is suppressed with used growth conditions. (f) Top end of the GaN nanocolumn.

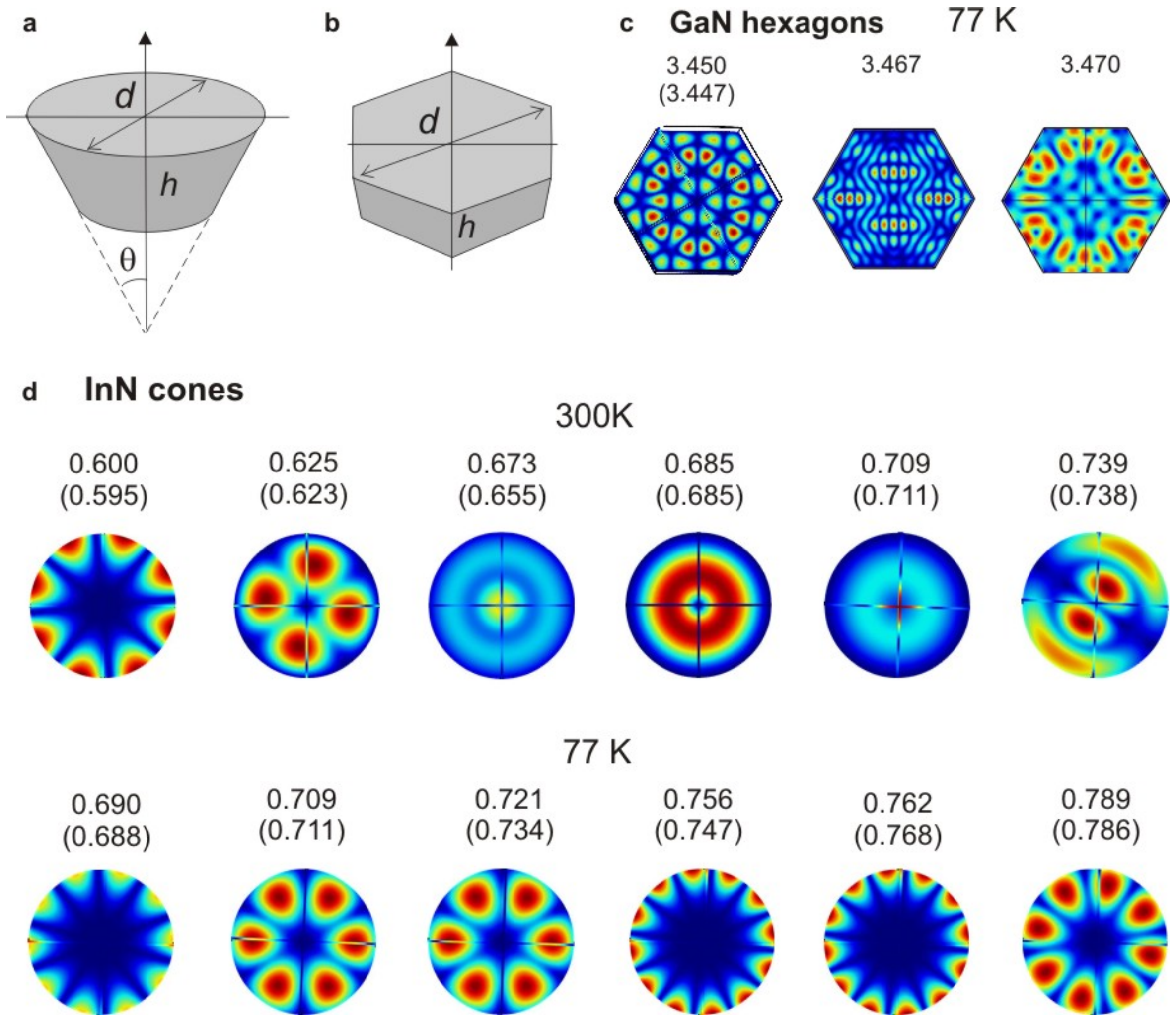

FIG. 2. Simulations of electromagnetic field intensity distributions and frequencies of basic quasi whispering gallery modes in GaN and InN cup-cavities. Two schematic presentations of cavity shapes were used: (a) a truncated cone for InN cavities; (b) a hexagonal prism for GaN cavities. The simulations were done for (c) the GaN cavity with a diameter *d*=1.2 μm at 77 K and (d) the InN cavity with *d*=2.2 μm at 77 K and 300 K. The theoretical mode energies are marked above the images; the experimental energies of the narrow emission lines are given in parentheses. For the energy 0.739 eV, similar mode turned by 90° is not shown for the sake of clarity. For the InN cavities, the simulations at two temperatures show the influence of refractive index variation on mode intensity patterns. They confirm the dominance of the azimuthal mode type at low temperatures, while the radial one is preferable at 300 K. In the obtained set for 300 K, the specific intensity pattern can be found, which provides the concentration of the electric field intensity in a subwavelength volume (see, e.g., the 0.673-eV mode). This effect is not pronounced in the cavities modelled by the hexagonal-prism, where the Fabry-Pérot modes dominate.
